# Sinks as a correctable source of ESBL contamination for patients in the ICU

**DOI:** 10.1186/cc11994

**Published:** 2013-03-19

**Authors:** I Wolf, P Bergervoet, W Van der Zwet, H Van den Oever, P Savelkoul, F Sebens

**Affiliations:** 1 Deventer Hospital, Deventer, the Netherlands; 2 VU Medical Centre, Amsterdam, the Netherlands

## Introduction

The incidence of patients carrying ESBL-positive bacteria in our ICU (12 in 780 admissions in 2011) was not considered problematic. However, routine cultures had identified ESBL-negative patients who had become colonized with ESBL strains during their ICU stay. Self-disinfecting siphons, preventing bacterial growth by antibacterial coating and intermittent heating, and biofilm formation by electromechanical vibration, were placed in all sinks in the ICU. The aim of the present study was to evaluate the effect of this intervention.

## Methods

An intervention study in a 12-bed ICU. The intervention involved placement of 19 self-disinfecting siphons (Biorec). All patients with an expected ICU stay of 2 days or more between January 2011 and December 2012 were studied. Samples of throat, sputum and rectum were taken at admission and twice weekly, and cultured for ESBLs. Between June 2011 and October 2011, sinks in patient rooms were cultured regularly for ESBLs. After the intervention in April 2012, multiple repeat cultures were taken. Whenever the species and antibiogram of bacteria cultured from patients and sinks matched, they were typed by AFLP.

## Results

**Figure 1:**
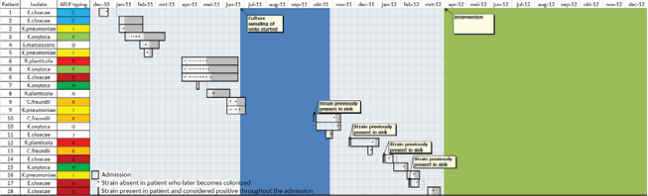
**Design and results of the intervention**.

*Before intervention *Multiple ESBL-forming strains were found in sinks of all patient rooms. Eighteen patients who were ESBL-negative on ICU admission became colonized with 11 different ESBL strains, that were present in sinks of their admission rooms (Figure 1). Four contaminations were proven by AFLP-tying. One patient died of ESBL-positive *E. cloacae *pneumonia. *After intervention *All sinks were negative for ESBL strains. No further patients became ESBL colonized during the ICU stay.

## Conclusion

Wastewater sinks were the likely source of ESBL colonization for 18 ICU patients. After placing self-disinfecting siphons there were no new ESBL colonizations in patients. This coincided with the disappearance of ESBL strains from all sinks.

